# Social determinants of health in Tunisia: the case-analysis of Ariana

**DOI:** 10.1186/1475-9276-8-9

**Published:** 2009-04-03

**Authors:** Habiba Ben Romdhane, Francis R Grenier

**Affiliations:** 1 National Public Health Institute, Tunis, Tunisia; 2 WHO Centre for Health Development, Kobe, Japan

## Abstract

**Background:**

Few research projects have analyzed how social determinants of health impact cities in North Africa. The sustained growth in these countries has nevertheless proven to exacerbate health disparities and create many social and economic inequalities. This strategic analysis examines selected social determinants of health in a major urban centre of Tunisia, identifies the most influential stakeholders able to influence equity/inequity, and reviews the accomplishments and need for action to foster health equity.

**Methods:**

This analysis was performed through a literature review and participatory research methods that included focus groups discussions and interview with key informants.

**Results:**

Access to health care, changes in lifestyles, housing issues and gender-related inequities are prime, socially-determined elements that affect health in Ariana.

**Conclusion:**

Recognition of emerging health issues is needed along with improved inter and intrasectoral coordination among stakeholders. The community-participatory approach used in this paper proved to be a useful scoping technique for this setting. A similar methodology could be used by other researchers as a first step toward health equity action at a city level.

## Background

Dramatic inequalities dominate global health today, with the conditions in which people grow, live, work and age having a powerful influence on their health [1]. Evidence suggests that this is particularly true in cities–where half of the world's population currently lives–and that the urban setting is a social determinant of health in itself [2]; living in cities increases exposure to unhealthy environments, disasters, climate change, violence and injuries, tobacco and other drugs, and epidemics [2] (Table 1).

**Table 1:** Participants in the community forum

**Government sector**	
*Education*	10
*Child care infrastructure*	2
*Youth and sport*	6
*Municipality*	30
*Media*	4
*Health Professionals*	7
*Other departments*	11
**NGOs and Volunteers**	
*Neighborhood Committees*	9
*NGOs*	10
**University researchers**	3

Tunisia, a low-middle income North African country, has seen in recent years a sustained economic, social and health development growth, rising from 0.516 on the Human Development Index in 1975 to 0.766 in 2005 [3]. Urbanization has also been constant, at an annual rate of approximately 3%: two-thirds of the population are now urban dwellers [4]. At the same time, Northern Africa has been the only developing region where the quality of urban life is improving, with the proportion of city dwellers living in slums decreasing by 0.15% annually. [4]. In Tunisia, this annual decrease has been estimated to be approximately 5% [5].

A strong social policy has played a major role in producing these positive results in Tunisia. Since 1969, national measures have included significant income transfers, starting with subsidies for basic food products; aid for indigent families and the unemployed in 1986; and in 2001, the creation of a national solidarity fund to support the development of basic infrastructures and services for low-income settings.

In terms of health care, both basic preventive and curative services are now available to almost the entire population, regardless of their socioeconomic status. As a result, the indicators of life expectancy, malnutrition and infant and maternal mortality have been consistently improved during the past decades. In spite of these dramatic improvements, gaps and inequities still exist.

In parallel with its rapid growth, Tunisia has experienced a sharp demographic and epidemiologic transition that has not been without negative effects. While controlling communicable diseases has so far been successful, Tunisians are now facing new problems such as hypertension, obesity, diabetes and tobacco use. Disparities in employment opportunities and housing conditions have also proved to have serious effects on the health status and general well-being of the population.

Despite the current consensus that the measurement of social determinants provides the needed evidence for further political action [6], to our knowledge, such an analysis has never been attempted in Tunisia, let alone on the local level. Tackling these underlying causes of poor health can contribute to improving health and health equity [7]. This study aims to identify the relevant social determinants–the causes of the causes–of health and assess their impact in an urban area of Tunisia.

## Methods

In 2006, with the support of the WHO Representative in Tunisia, the Epidemiology and Prevention Research Laboratory at the University of Tunis, the National Public Health Institute, the WHO Regional Office for the Eastern Mediterranean and the WHO Kobe Centre, a community-based intervention plan was developed for the city of Ariana.

This situation analysis, as the first part of this plan, draws principally on participatory research methods and on a review of relevant reports from the regional department of health, municipal government, national census, health surveys and official statistics. A community forum drawing participants from the government, academic and volunteer sectors took place in early 2007. A panel of experts was also established with faculty members from the University of Tunis, primary health care providers and an economist. The conduct of a round-table with participants from the government, NGOs, volunteers and academic institutions, joined by discussions with other key political figures, completed the information gathering process.

These consultations allowed for identifying current programmes and interventions aimed at improving urban health equity in Ariana and highlighted the barriers and difficulties experienced. This process also helped identify priority needs for action and health concerns for the local community.

Although it is not the biggest city in Tunisia, there were many reasons for selecting Ariana, which is located 40 km from the capital Tunis. Notably, Ariana is the most densely populated city of the country (5365 inhabitants/km), with a total population of 97 686 people [8]. It is also an ethnic melting-pot, fuelled by its long history as a focus of internal migration from all over the country. Urbanization has progressed dramatically in recent times at a rate of 6.5%, significantly higher than the national average of 1.2%. (Table 2)

**Table 2:** Demographic, economic and health indicators in Tunisia and Ariana

	Tunisia		Ariana
			
		*unit*	
Population	10.1 (2005)^b^	Millions	97 686 (2004)^a^
Population growth rate	1.2 (2005)^b^	%, annual	6.5 (2005)^a^
Density of population	57 (2003)^c^	Persons per km^2^	5 365 (2005)^a^
Infant mortality	20 (2005)^b^	Per thousand	22.1 (2005)^a^
Total fertility rate	1.9 (2005)^b^	Per woman	1.59 (2005)^a^
Life expectancy at birth			
Men	70 (2005)^b^	Years	74 (2005)^a^
Women	75 (2005)^b^	Years	75 (2005)^a^
Prevalence of obesity, adults			
Men	6.4 (1997)^b^	%	8.2 (2005)^a^
Women	22.7 (1997)^b^	%	24.4 (2005)^a^
Physicians	2.58 (2004)^b^	Per thousand	1.0 (2006)^d^
Adult literacy rate			
Men		%	96.0 (2005)^a^
Women		%	88.5 (2005)^a^
Total	74.3 (2004)^b^	%	

^a^Direction Régionale de la Santé (Ariana)

^b^World Health Organization, World Health Statistics, 2007

^c^FAO, 2004

^d^Government of Tunisia, Ministère de la Santé Publique, Direction des études et de la planification

Economic and social disparities in the city are pronounced, spatially segregating the neighbourhoods of the rich and poor. While unemployment is for the most part lower than the national average (6% vs. 15%), it continues to be a substantial problem affecting younger generations.

The genuine commitment of the local government for health promotion and environmental protection also justified the selection of this city for this project. Recent surveys and studies conducted in Ariana had highlighted the increasing burden of lifestyle-determined health conditions and the need for developing effective interventions.

Several motivations supported following a participatory model to pursue our analysis (Table 3). In general, previous research had notably proven the value of integrating "change agents", "catalysts", and "facilitators" to influence intersectoral policy and mobilize resources for health equity [2].

**Table 3:** Main benefits of the community participatory model

• All social groups feel concerned and participate in community matters;
• It addresses the true needs of community members, and there is better understanding of the causes and effects of problems;
• The solutions are adapted to community capacities and acceptable to all members, increasing their commitment to tackle the problems;
• The community is empowered, its dependency is reduced and there is an increased sense of ownership, self-responsibility, self-awareness and confidence in their own capabilities;
• People are interested in having a well-established and well-maintained project, building on existing local knowledge, resources and capacities;
• It is possible to generate community resources and reduces the overall costs and needed subsidies; this also improves the self-sufficiency and sustainability of the project.

A review of local health indicators and our initial discussions with the community led to the selection of access to health care, changes in lifestyles, housing issues and gender-related inequities as the key social determinants of health for this analysis.

## Results

### Particular health concerns in Ariana

In the last decade, numerous surveys carried out in Ariana by the National Public Health Institute (with the support of the World Health Organization) provided an important amount of data on noncommunicable diseases (NCDs) in this city, particularly on the prevalence of the major risk factors associated with cardiovascular diseases and diabetes. Evidence show that the incidence of NCDs in Ariana is increasing–representing almost 60% of the burden of disease, with cardiovascular pathologies being the major killers (Tables 4 and 5).

**Table 4:** Prevalence of risk factors in Ariana

	Male	Female
		
	%	%
*Risk factor*		
Use of tobacco	46.9	5.9
Hypertension	35.9	46.2
Obesity	8.2	24.4
Diabetes	13.6	15
High cholesterol	8.7	18.2

Source: [18]

**Table 5:** Principal causes of death in Ariana

	Male	Female
		
	%	%
*Cause of death*		
Cardiovascular diseases	28.3	24.2
Cancer	16.1	19.0
Injuries	12.5	7.4
Respiratory diseases	13.0	6.4

Source: [19]

Apart from tobacco, women are afflicted by a higher prevalence of all other NCD risk factors, with in addition poor awareness and control of hypertension and diabetes. A survey conducted with secondary school youth [9] showed that dietary habits are also influenced by gender, with female reporting the worst behaviours.

The level of physical activity during leisure time is very low, especially among women. Slightly less than one third of female secondary school youth report performing physical activity regularly, a figure that is significantly less than what is reported by males [9]. This can at least partly explain the 20% prevalence of obesity in secondary school girls. There is a lack of evidence on the amount of physical activity performed as part of their work or for commute. Certainly, cars are increasingly favoured over non-motorized forms of transportation such as walking or cycling. Insufficient infrastructure for cycling, the lack of pedestrian paths and few sport centres can partly explain this data.

Use and exposure to tobacco is another crucial municipal concern. Despite the enactment of a law that banned smoking in public areas, second-hand smoke exposure is widespread in restaurants and coffee places–not to mention hospitals–where health personnel often smoke. Besides living in homes where they are exposed, there is limited enforcement of tobacco sales to children and smoking by teachers in the school environment–and sometimes in classrooms–is common. Community surveys performed during this study also highlighted the lack of information and low access to tobacco cessation programmes and methods.

Consumption of alcohol by youth is another problem, and 28.6% of schoolboys and 7.1% of girls have reported drinking in the past [9].

### Health care services in Ariana

A number of private and public stakeholders influence health directly or indirectly through their effect on social determinants of health.

Health promotion is characterized by a variety of responsibilities shared between the regional offices of the Ministry of Health and of the Ministry of Social Affairs, the municipal environmental office and in some way several other departments. As Tunisia has a strong central government, allocations for health promotion are decided by the Ministry of Health, which therefore set most of the priorities.

The programmes overseen by the municipality include environment protection (solid waste, water and sanitation, transport), administration of parks and walking areas, and tobacco control. Ariana, commonly called the "city of roses", is one of the most committed to environmental protection. The municipality also contributes to the rehabilitation of poor neighbourhoods and manages cultural and social venues and childcare services. The city coordinates many of its activities with regional and local agencies including the Regional Ministry of Infrastructure, the National Society of Electricity and Gas (STEG), the Housing Rehabilitation and Renovation Agency (ARRU) and other NGOs and business organizations.

The Regional Department of Health develops annual health programmes based on the evolving epidemiological and socioeconomic situation. The RDH organizes "Health Caravans"–mobile units staffed by a cardiologist, an ophthalmologist and a gynaecologist–to provide speciality health care to those in need. The Department also oversees mental, sexual and reproductive health programmes and provides assistance for students with disabilities, social problems and learning difficulties; it also supports the optometry and dentistry academic programmes.

Primary health care centre staff play an important role in reducing low and middle-class populations' health problems, not only with concrete initiatives but also by advocating for improved quality of care and better drug coverage for underprivileged patients. Health care professionals have a central role in promoting healthy lifestyles and providing information on the consequences of unhealthy behaviours. According to a survey conducted among young people in Ariana, 36% of health education is carried out by medical staff [9]. Nurses play a particular role in helping and counselling isolated and elderly patients.

Ariana is home to many private sector businesses. The Regional Department of Social Affairs (RDSA) contributes to local health by undertaking occupational health functions. Many workers are nevertheless vulnerable because of poor job security and need specific programmes. The RDSA also contributes to health equity through health insurance programmes and by the disbursement of direct monetary aid to the poorest members of the population.

School is an important channel to promote healthy lifestyles not only to students but also to their families and communities. At the university level, there are also several groups involved in health promotion (tobacco control, STD prevention, nutrition and physical activity).

The role of Nongovernmental Organizations is currently limited to information, counselling and a certain amount of support for disadvantaged families. A consumer association is very active and its members promote health through specific booklets, pamphlets and informative sessions on healthy lifestyles. In the future, NGOs should be more involved in identifying the priorities of exposed populations and interventions, as several of them have been able to interface with groups that are more difficult to reach, such as women and disadvantaged populations.

Volunteers have not been involved in community health activities so far, but have expressed their interest. Members of the university health clubs and groups of women and retirees were the most enthusiastic.

Media have a crucial role in disseminating information on healthy lifestyles and initiatives related to healthy urbanization. The city also publishes a newsletter dealing with lifestyle issues. These are important channels for health promotion.

Several universities and institutes within the city have in the past conducted community-based intervention research, while others participated in the dissemination of health-related research, training and advocacy.

Ariana has many representatives and spokespersons who could be involved in advocacy for healthy environments, potentially promoting new legislation related to better food and against tobacco.

### Access to health care

While the city of Ariana is not one of the poorest communities in the country, it does demonstrate deep population stratification according to economic, social and cultural status. A high socioeconomic status population (among the highest in the country) mixes with a middle class and a very poor population with increasing health needs.

Socioeconomic status is an important factor in determining access to sport facilities, health information, healthy food, housing and health care. This affects the management and prevention of NCDs, the most prevalent health problem in the community. With the current prevalence of NCDs and the limited resources for primary health care services, many patients are unlikely to be receiving the treatment they need.

Women's access to health services is limited to family planning and contraception. Use of contraception is widespread and the fertility rate is low, even in populations of low socioeconomic status; there is nevertheless an unsatisfactory record with the other components of reproductive health, including screenings for breast and cervical cancer. Sexual health and sexuality issues are not adequately addressed and quality of care is a critical problem reported by our participants.

A review of local health services policies has identified that some benefits are provided to extremely needy persons–families, elderly and disabled–including completely free medical assistance and partially subsidized programmes. Nevertheless, too many cannot get the help they need because they do not meet the requirements (age, unmet qualifying period of work, not recognized disability) to enrol into these programs.

The availability of drugs, especially for chronic diseases such as diabetes and hypertension, is still a major problem for a large part of population. Being confined to primary health care centres–where the availability of drugs is often limited to one or two classes and where shortages often occur–many people are unable to achieve optimal control of their health conditions.

The management of NCDs combines all the difficulties that could possibly be encountered by patients. These diseases have a marked effect on differential access to care and treatment (drugs, access to specialists and medical examination) for groups with modest income.

### Lifestyle environment

Unhealthy lifestyles, stress, tobacco use, deficient diets, lack of physical activity, obesity and diseases related to environmental pollution are giving rise to various health problems, causing a great burden of morbidity and mortality.

Tunisia is now facing an epidemic of obesity linked closely to the popularity of fast-food and lifestyle transitions. Food consumption patterns and dietary habits have changed markedly during the last few decades, with an increase in per capita energy and fat intake, and a decrease in physical activity (replaced with long periods spent watching television and surfing the internet).

The westernization of diets is noted as a major concern in the food environment. Many large stores offer all kinds of unhealthy food such as sweets, soda, and salty snacks. Advertisements for unhealthy food are also increasing dramatically on TV and in transport facilities, targeting children in particular. Young people are rapidly adopting behaviours linked with unhealthy eating habits and lower levels of physical activity at school or during leisure time. It is probable that this rapid evolution towards a westernized way of life will continue with the urbanization that is currently underway. The rapidity and brutality of these changes endanger health by negatively affecting values, lifestyles, diet and social organization.

Many healthcare workers expressed their concerns about how social transformations (female entry to the labour market, situation of young people), new lifestyles and changes in socioeconomic conditions (displacement from rural areas, loss of employment, etc.) affect the pattern of diseases, particularly for younger people and women.

Urban residents perceive themselves as "more stressed than rural people" and it is worth noting that this stress is related to lifestyle and to socioeconomic factors. Food and eating habits appear to play an important role in stress management, in particular among women. The latter frequently reported stress-related eating patterns that could at least partially explain the levels of obesity in this population.

To stem the rising tides of lifestyle-related diseases, comprehensive approaches for improving nutrition and physical habits that target both individuals and the population are required [10,11].

### Housing

Promiscuity, domestic waste and sewage management, pollution and infestation by vectors of diseases are critical problems that are particularly present in lower-income areas of the city. There is a lack of suitable parks and green spaces available for leisure activities, leaving only cafes–whose access is often restricted to men. There are also very few facilities for the elderly and disabled.

This unhealthy environment is also a threat regarding the transmission of communicable diseases. Several people also attribute their child's school difficulties to their housing conditions. In addition, transport issues in commuting cause stress for many workers, especially women.

In many neighbourhoods, severe issues are brought by people moving in from the countryside, maintaining many of their unhealthy habits (e.g. keeping large animals in the house)–but not their healthful traditional ways of cooking and social organization–while at the same time adopting some other unhealthy urban behaviours such as inactivity and poor dietary habits.

There is a lack of data available on the health issues associated with the current housing environment in Ariana. A baseline survey is currently being planned and promises to fill this gap.

### Gender equity

In several countries (especially in the Eastern Mediterranean region), the lower social status of women is a key underlying social determinant of health: gender-based differences in access to health, in mortality and morbidity have been found in many studies [12-14].

In Tunisia, the principle of equality between men and women was clearly and expressly stipulated in the law as early as 1956. This longstanding gender policy is bearing fruit and the legal status of women in Tunisia has been one of the strongest and most liberal in the Arab world [15,16]. This has helped to make important achievements in reducing the gender gap in education and resulted in increased literacy and school enrolment rates for women.

Several measures have also been taken to enhance the participation of women in the public arena, including introducing a quota of 20% for female candidates in political parties. They now represent 22.7% of the members of the Chamber of Deputies; 27.4% of all elected official at municipal councils; and 26% of the ruling party members. Nevertheless, women appointees represent only 15% of all the cabinet positions; 15% in the Chamber of Advisors; and 11% at the Economic and Social Council. Women do have a higher presence in the media (about 35%).

In 1964, Tunisia adopted an ambitious family planning programme with health education being promoted throughout the country; family planning and maternal-child health services were also combined in basic health care centres and made more readily available in remote rural areas, including through mobile units. This proved successful–particularly in removing cultural and economic barriers–and remains a good model for developing countries. Women are present in different community health structures as doctors, nurses, midwives, and social workers, where they have a significant role in community health promotion.

It is clear that Tunisian family and traditional social roles are undergoing a deep change. Nowadays, most women share the responsibilities in the household–although not always equitably–with their partners. A critical element for working women remains the availability of good childcare facilities. Educated mothers understand the importance of early childhood education and are increasingly using such services to assist with childcare.

Health indicators have improved in Tunisia for both sexes but the greatest achievements are probably those related to family planning and maternal health care. Tunisian women have indeed achieved tremendous advancement in contraception issues, where they are now empowered to take decisions by themselves.

Despite these improvements in relation to gender disparities, involvement in the decision-making process is still profoundly linked to economic status, and poor women have difficulty in making their voices heard. Women also continue to be affected by other social and economic factors, such as work conditions that are tedious and physically exhausting.

Another area that needs intervention is domestic violence. Although there is no official data that highlights the magnitude of this problem in Tunisia, our focus group female participants reported that they believe that domestic violence is increasing, and men believe that violence is a consequence of their counterparts' emancipation. Could the freedom that women acquire constitute a source of frustration to their partners? Difficulties encountered with divorce proceedings and cultural issues, including social values that tolerate violence against women, are other explanations for that phenomenon.

The significant epidemiological transition, including the higher prevalence of the major cardiovascular diseases risk factors in women, is certainly an important challenge for the coming years in regard to women's health. Sub- heading for this section

## Discussion

This analysis will be used as a reference for the city of Ariana in designing its project on healthy urbanization, as it identifies other inadequacies, and as a baseline in assessing its interventions based on social determinants of health. In the local context, further action to optimize the impact of those determinants will require continuous research, community mobilization, advocacy and leadership. We have highlighted what has been achieved so far and what is now needed to foster equity in Tunisia in terms of legislation and policies, capacity building, management, and commitment.

Ariana, like many cities in Tunisia, is now facing a new challenge: the emergence of NCDs as a result of the growth of lifestyle-related risk factors. As in the case of Ariana, previous research has highlighted the particular importance of lifestyle in health [17]. There is a need to further study how the intra-city inequities impact on transition in lifestyle and behaviours that could increase the major health risk factors.

Several studies have highlighted the need to be prepared to face emerging health problems. The limited resources currently available hint that many patients would likely not receive the treatments they need in such circumstances. Better planning and an equitable pooling of resources are needed.

Stakeholders have the ability to modify the impact of the key social determinants of health but many are not aware of the effects of social determinants on health and of their role in equity. In our discussion groups, some appeared quite knowledgeable about emerging diseases and the impact of urbanization on health status, but only a few could perceive the impact of social determinants.

The sharing of responsibilities across multiple stakeholders should not create major conflicts, and the coordination and partnership challenges are less daunting than those imposed by the economic, social and epidemiological transition. It is however very common for programmes in the same community to be uncoordinated, which leads to gaps and overlaps in service provision.

At the same time, in order to improve equity in access to health care and given the spread of emerging chronic diseases, a profound reform of the health system is urgently needed; this is particularly required for vulnerable and disadvantaged segments of the society. In addition to investment, the changes should include reorganisation of health services to reinforce the role of general practitioners and a new basic curriculum for medical doctors and health personnel to enable them to shift from a "diseased-centred" to a "patient-centred" paradigm.

## Conclusion

Our analysis proved to be an effective initiating approach for assessing the impact of urbanization in a rapidly developing city of a Northern African country facing a crucial epidemiological transition. It also identified several opportunities to optimize the impact of social determinants of health not only in our setting but for other communities.

There are pronounced economic and social gaps in the city, with poverty being concentrated in a few neighbourhoods. While we recognize this problem, we also acknowledge the need for further research, measure and assessment of the inequities across the city to ensure a complete understanding of the mechanisms that create these disparities.

Stakeholders need to reconsider their roles and strategies and increase their level of intersectoral collaboration and coordination. In spite of the added complexity at the local level, this should include deciding on shared policies and common goals.

This initiative fulfilled one of the recommendation identified by the Commission on Social Determinants of Health in its final report [20], that acknowledging that there is a problem is a vital step to ensure follow-up action. In order to address the Commission's recommendation in its entirety, a new project to assess intra-city inequities across various policy domains will be initiated in Ariana in the next few months.

## Competing interests

The authors declare that they have no competing interests.

## Authors' contributions

HBR carried out the participatory research methods. FRG participated in the design of the study and coordination of the project. All authors participated in the drafting of the manuscript, read and approved its final version.

## References

[B1] World Health Organization (2007). Achieving Health Equity: from root causes to fair outcomes. Geneva.

[B2] Mercado S, Havemann K, Sami M, Ueda H (2007). Urban Poverty: An Urgent Public Health Issue. Journal of Urban Health.

[B3] United Nations Development Programme (2007). Human Development Data New York.

[B4] UN-HABITAT (2005). Financing Urban Shelter.

[B5] UN-HABITAT (2001). Statistical Overview – Tunisia Nairobi.

[B6] Kelly MP, Bonnefoy J (2007). Developing an evidence base for political action.

[B7] Feachem RGA (2000). Poverty and Inequity: A Proper Focus for the New Century. Bulletin of the World Health Organization.

[B8] Institut National de la Statistique (2006). Annuaire Statistique de la Tunisie [in French]. Tunis.

[B9] Bougatef S, Skhiri H, Elkhedim H, Bergaoui N, Ben Romdhane H (2006). Prévalence et facteurs associés a l'obesité chez l'enfant en âge préscolaire dans le gouvernorat de l'Ariana: résultats d'une enquête realisée en 2006 [in French].

[B10] Swinburn B, Egger G (2002). Preventive strategies against weight gain and obesity. Obesity Reviews.

[B11] World Health Organization (2003). Diet, nutrition and the prevention of chronic diseases. Geneva.

[B12] Fikree FF, Pasha O (2004). Role of gender in health disparity: the South Asian context. British Medical Journal.

[B13] Yount KM (2003). Provider bias in the treatment of diarrhea among boys and girls attending public facilities in Minia, Egypt. Social Science & Medicine.

[B14] Mohammadi MR, Ghanizadeh A, Rahgozart M, Noorbala AA, Malekafzali H, Davidian H (2005). Suicidal attempt and psychiatric disorders in Iran. Suicide Life Threat Behav.

[B15] Centre de Recherches, d'Etudes, de Documentation et d'Information sur la Femme (1994). Femmes de Tunisie – Situation et Perspectives [in French] Tunis.

[B16] Centre de Recherches, d'Etudes, de Documentation et d'Information sur la Femme (2002). Les Femmes en Tunisie [in French] Tunis.

[B17] Robertson A, Brunner E, Sheiham A, Marmot M, Wilkinson RG Food is a political issue. Social Determinants of Health.

[B18] Ben Romdhane H, Skhiri S, Bougatef DG, Ben Azouz H, Achour N (2005). Surveillance des maladies cardiovasculaires en Tunisie: méthodologie et principaux résultats [in French]. Tunisie Médicale.

[B19] Ben Romdhane H, Haouala H, Belhani A, Drissa H (2005). [Epidemiological transition and health impact of cardiovascular disease in Tunisia]. Tunisie Médicale.

[B20] WHO (2008). Closing the gap in a generation. Geneva.

